# Characterization of psychrotrophic and thermoduric bacteria in raw milk using a multi-omics approach

**DOI:** 10.1099/mgen.0.001311

**Published:** 2024-11-06

**Authors:** Xue Qin, Jingqi Cheng, Yue Qiu, Ning Guan, Tanushree B. Gupta, Shuyan Wu, Yujun Jiang, Xinyan Yang, Chaoxin Man

**Affiliations:** 1 Key Laboratory of Dairy Science, Ministry of Education, College of Food Science and Engineering, Northeast Agricultural University, Harbin 150030, PR China; 2 Center for Dairy Safety and Quality, National Center of Technology Innovation for Dairy, No.1 Jinshan Road, Jinshan Development Zone, Hohhot, PR China; 3 AgResearch Ltd, Hopkirk Research Institute, Cnr University Ave and Library Road, Massey University, Palmerston North 4442, New Zealand

**Keywords:** metagenomics, metaproteomics, psychrotrophic and thermoduric bacteria, raw milk

## Abstract

Psychrotrophic and thermoduric bacteria are the main reasons for the spoilage of dairy products. This study aims to address the composition and function of psychrotrophic and thermoduric bacteria in eight groups of raw milk samples obtained from Heilongjiang Province and Inner Mongolia (China). Microbial enumeration showed an average total bacterial count of 4.63 log c.f.u. ml^−1^ and psychrotrophic bacterial counts of 4.82 log c.f.u. ml^−1^. The mean counts of mesophilic and thermophilic thermoduric bacteria were 3.68 log and 1.81 log c.f.u. ml^−1^, respectively. Isolated psychrotrophic bacteria (26 genera and 50 species) and mesophilic thermoduric bacteria (20 genera and 32 species) showed high microbial diversity. Through metagenomic and proteomic analyses, significant disparities in the concentration and community structure of psychrotrophic and thermoduric bacteria were observed among different locations. A large number of peptidases were annotated by metagenomics, which may result in milk spoilage. They mainly come from some typical psychrotrophic and thermoduric bacteria, such as *Chryseobacterium*, *Epilithonimonas*, *Pseudomonas*, *Psychrobacter*, *Acinetobacter, Lactococcus, Escherichia* and *Bacillus*. However, the main proteins detected in fresh raw milk were associated with bacterial growth, reproduction and adaptation to cold environments. This investigation provides valuable insights into the microbial communities and protein profiles of raw milk, shedding light on the microbial factors contributing to milk deterioration.

**Impact Statement**

Psychrotrophic and thermoduric bacteria in raw milk are considered to be the main factors to affect milk quality. In this study, microbial contamination in raw milk samples from Heilongjiang Province and Inner Mongolia (China) were investigated. *Pseudomonas*, *Lactococcus* and *Acinetobacter* were dominant psychrotrophic bacteria. *Enterococcus*, *Enterobacter*, *Lactococcus* and *Bacillus* were the most frequently isolated mesophilic thermoduric bacteria. *Deinococcus geothermalis* and *Staphylococcus pasteuri* were thermophilic. Two groups of typical samples were selected to explore the relationship between microbial structure and proteins. A large number of peptidases were annotated by metagenomics, such as PepA aminopeptidase, aminopeptidase P3, HtpX peptidase and AlgW peptidase, but interestingly, they were not detected by metaproteomics. Most expressed proteins in fresh raw milk were related to bacterial growth, reproduction and adaptation to cold environments rather than proteases or lipases. This paper offers valuable insights to better understand the potential impact of the microbiota influencing milk quality from three aspects of culture, metagenomics and metaproteomics, providing a comprehensive view for further study of enzymes produced by psychrotrophic and thermoduric bacteria in raw milk.

## Data Summary

All metagenomic raw sequences have been deposited to the National Center for Biotechnology Information (NCBI) sequence read archive (https://www.ncbi.nlm.nih.gov/sra) under accession numbers SRR30211375 to SRR30211380. The metaproteomic data have been deposited to the ProteomeXchange Consortium (https://proteomecentral.proteomexchange.org) via the iProX partner repository [1,2] with the dataset identifier PXD054735.

## Introduction

Raw milk offers an ideal environment for micro-organisms to thrive, given its abundant nutrients, nearly neutral pH and high water activity. Consequently, milk contamination by micro-organisms remains an ongoing concern. Throughout milking, storage, transportation and processing, raw milk can get contaminated with various micro-organisms. Even when stored at controlled temperatures, the presence of spoilage bacteria remains persistent as a concern causing milk contamination [3]. Psychrotrophic and thermoduric bacteria are common spoilage bacteria. Psychrotrophic bacteria are ubiquitous organisms capable of growing at below 7 °C, in spite of their high optimum growth [4]. Thermoduric bacteria are a group of micro-organisms that are resistant to heat treatment in traditional processes [5].

Microbial diversity in raw milk has been extensively studied in recent years. Research has identified various psychrotrophic and thermoduric bacteria, such as *Pseudomonas*, *Flavobacterium*, *Chryseobacterium*, *Acinetobacter* and *Bacillus*, as the main culprits behind milk spoilage [6]. These bacteria can contaminate milk from sources such as water, soil, udder and teat surfaces and farm storage tanks, pumps, pipes and processing equipment. Conventional heat treatment effectively eliminates psychrotrophic bacteria, but it may not inactivate the heat-stable proteases they produce. As a result, residual proteases persist and continue to degrade the milk quality during storage. The residual proteases induce a bitter flavour and gelation [7]. Lipases produced by some psychrotrophic bacteria are thermostable, and the residual lipases hydrolyze fat and the released fatty acids can cause off-flavours in milk [8]. Therefore, the analysis of spoilage microbial diversity in unpasteurized milk holds significance.

The microbial communities present in raw milk exhibit a richness and complexity that exceeds the ability to comprehensively characterize based on traditional culture techniques. High-throughput sequencing based on the highly variable region of 16S rRNA is popularly used in the field of dairy microbiology [9]. Although high-throughput sequencing can quickly and accurately identify the species and abundance of micro-organisms, it can only annotate micro-organisms at the genus level and cannot meet the needs of more in-depth analysis. Metagenomic sequencing technology has emerged as a powerful tool for in-depth analysis of microbial communities at the genetic and functional levels. Random fragmentation, reassembly and annotation of the microbial genome allow for the investigation of microbial evolution, examination of gene functionalities and species-level or strain-level annotations.

There is a dynamic interaction between bacteria and the milk system, where the bacterial community correlates with specific proteins/peptides and fatty acid profiles [10]. Our research also integrated metaproteomics (proteomics performed in bulk population of cells) to conduct a more comprehensive investigation of the microbial dynamics and various gene expressions in bacteria present in raw milk, which could shed light on the underlying reasons for raw milk deterioration. Metaproteomics gathers data concerning the proteins present and the biochemical pathways employed by the microbiota in the given sampling circumstances [10], offering unique insights not achievable through metagenomics. Proteomics based on data-independent acquisition (DIA) in four dimensions (4D) is a highly promising technological approach, with the potential to achieve enhanced coverage of protein samples [11]. Metaproteomics based on 4D-DIA technology plays a vital role in comprehensively analysing microbial proteins within the research environment. This technique is essential for gaining insights into the functional characteristics and behaviour of spoilage micro-organisms found in raw milk.

The main objective of this research was to analyse the composition of bacterial communities and microbial proteins in raw milk samples obtained from Heilongjiang and Inner Mongolia Province in China. This was achieved by utilizing advanced techniques such as Illumina-based metagenomic sequencing and 4D-DIA metaproteomics. The aim of this investigation was to evaluate the protease-producing and lipase-producing abilities of micro-organisms in fresh milk and gain a better understanding of the potential quality deterioration of micro-organisms in raw milk. The findings will provide insights valuable for developing new strategies to improve dairy product preservation and ensure the production of premium-quality dairy items.

## Methods

### Raw milk sampling

Twenty-four raw milk samples from milk tanks were collected from two different commercial dairy farms located in the Heilongjiang (HLJ) and Inner Mongolia Province (NMG) of China from August 2022 to April 2023. The milk samples were divided into eight groups of samples, and three parallel samples of each group were from the same tanks (commingled milk harvested from the same herd). The detailed sampling time is listed in Table 1. Three parallel samples (one group sample) were collected for every sampling time. Under conditions of strict sterility, the collected samples were obtained and promptly transported to the laboratory at a temperature of 4 °C for immediate analysis.

**Table 1.** The details of the sampling

Dairy farm	Sampling time
HLJ	August 2022, September 2022 (A), October 2022, March 2023 and April 2023
NMG	August 2022, September 2022 (B) and March 2023

### Bacteria enumeration and isolation

To determine the total bacterial count (TBC), a volume of 25 ml from each raw milk sample was utilized in the testing process. The samples were serially diluted tenfold to 10^5^ with 0.1% sterile protein saline (w/v), 100 µl for each dilution, and plated on plate counting agar (PCA, Qingdao Hope Bio-Technology Co., Ltd., China). The plates were incubated at 37 °C for 48 h. The milk samples for psychrotrophic bacterial counts (PBCs) were diluted tenfold to 10^5^, and each dilution was cultured on milk powder count agar (MPC, Qingdao Hope Bio-Technology) at 7 °C for 10 days according to a previous method [12]. The thermoduric bacteria were enumerated after subjecting the milk sample (5 ml) to a pasteurization simulation condition (62.8±0.5 °C for 30 min) followed by immediate refrigeration at 10 °C for 10 min. Then, the samples were serially diluted tenfold to 10^3^, and each dilution (100 µl) was spread on PCA in triplicates. The mesophilic and thermophilic thermoduric bacteria were cultured at 35±1 °C and 55±1 °C for 48 h, respectively [13]. All the plates were incubated in aerobic constant temperature incubators. The standard bacterial count of each sample was shown as log c.f.u. ml^−1^ after incubation. The traditional culture-based method, as a gold standard, is widely used to isolate bacteria [12]. Randomly, 50–60 colonies with different morphologies were selected from the counting plate (30–300 colonies) to ensure that one or more isolates represent different colony morphologies. The culture conditions were as follows: 25 °C for psychrotrophic bacteria, 37 °C for mesophilic thermoduric bacteria and 55 °C for thermophilic thermoduric bacteria. In total, except for non-culturable colonies, 356 isolates were collected for 16S rRNA gene sequencing.

### 16S rRNA gene amplification

Psychrotrophic, mesophilic and thermophilic thermoduric bacteria were cultured in Luria Bertani broth (LB, Qingdao Hopebio Technology, China) at 25 °C, 35±1 °C and 55±1 °C for 24 h, respectively. The extraction of genomic DNA was carried out following the one-step water bath method [14]. DNA concentration was not less than 20 ng µl^−1^, and A260/A280 was between 1.8 and 2.0. The genomic DNA was employed as a template for performing PCR targeting the 16S rRNA gene, employing the universal primers 27F (5′-AGAGTTTGATCCTGGCTCAG-3′) and 1492R (5′-TACGGCTACCTTGTTACGACTT-3′). PCR amplification and agarose gel electrophoresis (AGE) verification were executed following the protocols established by Yang *et al.* [12]. The amplified products were sent to BeiJing RuiBiotech Co., Ltd. (Beijing, China) for Sanger sequencing (3730xl DNA analyzer). Strains were identified by blasting sequencing results from the NCBIBLAST website (http://www.ncbi.nlm.nih.gov/bLAST/).

### Metagenomics

Two groups of milk samples from HLJ (A) and NMG (B) (three parallel samples each), collected in September 2022, were chosen for further omics analysis (Table 1). The replicate samples came from three samples in the same groups. The Mag-Bind Soil DNA Kit (OMEGA Bio-tek, GA) was utilized to extract the genomic DNA from the milk samples. The quantity and quality of extracted DNAs were measured using a Qubit 4 Fluorometer, with WiFi: Q33238 (QubitAssay Tubes: Q32856 and Qubit 1X dsDNA HS Assay Kit: Q33231) (Invitrogen, USA) and AGE, respectively. The extracted microbial DNA was processed to construct metagenome shotgun sequencing libraries with insert sizes of 400 bp by using Illumina TruSeq Nano DNA LT Library Preparation Kit. Each library was sequenced by Illumina NovaSeq platform (Illumina, USA) with PE150 strategy at Personal Biotechnology Co., Ltd. (Shanghai, China). A sequencing approach employing paired-end reads, with a target read length and insert size of 150 bp, was employed. Cutadapt (v1.2.1) was utilized to screen the paired-end raw data, obtaining high-quality CleanData for subsequent metagenomic analysis. The microbial composition spectrum was determined at both genus and species levels, identifying species that displayed significant differences between groups A and B based on the criteria of ⌈Log2(Fold change value)⌈ >1 and *P<*0.05 by metagenomeSeq method [15]. Alpha and beta diversities of the two groups of milk samples were computed by QIIME2 (v1.9.1) and R v.4.2.0 software (http://www.R-project.org) according to the species composition spectrum. In order to analyse and identify the dissimilarities between the two groups of milk samples, linear discriminant analysis effect size (LEfSe) was employed. The use of principal coordinate analysis (PcoA) plots allowed for the visualization of the beta diversity observed within the samples. Statistical methods were then used to select the core function and dominant species. To provide functional annotations to the sequencing data, databases namely the Kyoto Encyclopedia of Genes and Genomes (KEGG) (v2020_10_20) [16], the Evolutionary Genealogy of Genes: Non-supervised Orthologous Group (EggNOG) (v5.0), KEGG Orthology (KO) (v2020_10_20), Carbohydrate-Active enZymes Database (CAZy) (v2021_09_24) [17], Swissprot (v2021.9.24) and MEROPS (v2021.9.24) were utilized. Furthermore, the functionality of genes and proteins within the raw milk samples was analysed using the above database.

All bioinformatic and statistical analyses were conducted using R software version 4.2.0 (http://www.R-project.org). The raw sequencing data for each sample were sorted, and the sequencing volume along with the proportion of high-quality bases was calculated to assess the overall quality of the sequencing. Subsequently, the raw data were screened and filtered using fastp (version 0.20.0), which involved removing 3' end-joint sequences and sequences shorter than 50 bp and those containing ambiguous bases. Minimap2 software (version 2.24-r1122, https://github.com/lh3/minimap2) was used to compare the above effective sequences and host sequences and then discarded host matching sequences in order to remove as many contaminating host sequences as possible, resulting in a CleanData set (i.e. a set of target sequences). Kraken2 (version 2.0.8-beta) [18] was employed on the CleanData, utilizing microbial genome data from the National Center for Biotechnology (NCBI)’s RefSeq genome database as a reference to construct a database, with the confidence level set to 0.5 for annotation analysis. Non-target sequences were filtered out and subsequently spliced. Splicing was performed using MEGAHIT (v1.2.9) [19] to retain contigs with a length of at least 300 bp. Finally, all contig sequences were merged. Using MMseqs2 software (v 13–45111), redundancy within the contig sequence set was eliminated based on a similarity threshold of 95% and a coverage of 90%, resulting in a non-redundant contig set. MetaGeneMark software (v3.26) (http://exon.gatech.edu/GeneMark/) was then employed to predict prokaryotic microbiology and macro genome sequences, identifying ORFs and predicting coding regions to obtain the corresponding gene and protein sequences. The taxonomy module of MMseqs2 (v 13–45111) was utilized to compare the non-redundant protein sequences against the NCBI nr database (version 2021.10.11, corresponding to the download date), with the sensitivity parameter set to 5.7 and the lca-mode set to 4, allowing for the selection of species information with the highest alignment score as the representative species information for the protein gene sequences.

### 4D-DIA metaproteomic analysis

To prepare the raw milk samples for analysis, they were mixed with SDT (SDS and DTT) lysis buffer containing 4% SDS, 100 mM Tris-HCl at a pH of 7.6, and then boiled for 15 min. The protein component of each sample was collected through centrifugation at 14 000 ***g*** for 40 min. The collected protein was quantified using the BCA Protein Assay Kit from Bio-Rad (USA). Next, 20 µg of protein from each sample was mixed with the appropriate 5× loading buffer and boiled for 5 min. The proteins were then separated using a 4–20% SDS-PAGE gel with a constant voltage of 180 V for 45 min. To visualize the protein bands, Coomassie Blue R-250 staining was used. For further analysis and quality control purposes, an equal aliquot from each sample was combined into one sample. This pooled sample would be used for data-dependent acquisition (DDA) library generation.

Each sample was treated with DTT, a detergent known as detergent dithiothreitol, at a final concentration of 10 mM. The mixture was then blended at 600 r.p.m. for 1.5 h at a temperature of 37 ℃. Subsequently, to block reduced cysteine residues, iodoacetic acid (IAA) was added to the mixture at a final concentration of 20 mM. The samples were left to incubate in darkness for 30 min, following which they were cooled to room temperature. Next, the samples were purified and concentrated by transferring them to 10 kDa filters. To achieve this, the filters were washed three times with 100 µl of UA buffer (consisting of 8 M urea, 0.1 M Tris-HCl and 50 mM dithiothreitol) and twice with 100 µl of 25 mM NH_4_HCO_3_ buffer. Subsequently, trypsin was introduced into the samples in a 1 : 50 trypsin to protein (wt/wt) ratio. The samples were incubated at 37 ℃ for 15–18 h (overnight), resulting in the collection of peptides as a filtrate. The desalting of each sample’s peptides was performed using C18 Cartridges (Empore SPE Cartridges C18), with a bed I.D. of 7 mm and a volume of 3 ml, sourced from Sigma. These peptides were then concentrated via vacuum centrifugation and reconstituted in 40 µl of 0.1% (v/v) formic acid. The estimation of the peptide content was accomplished by utilizing UV light spectral density at 280 nm. To conduct DIA experiments, indexed retention time (iRT) calibration peptides were added to the samples.

The TIMSTOF (Trapped Ion Mobility Spectrometry Time of Flight) mass spectrometer (Bruker) was used to analyse all samples of fractions. It was connected to an Evosep One system liquid chromatography system (Denmark) to ensure accurate results. A dynamic exclusion of 24.0 s was implemented to avoid repetitive measurements. The ion source voltage was set at 1500 V, while the temperature was maintained at 180 ℃. A dry gas flow rate of 3 l min^−1^ was used. Ion mobility scanning was performed within the range from 0.75 to 1.35 Vs cm^−2^, followed by eight cycles of PASEF (Parallel Accumulation–Serial Fragmentation) MS/MS scanning. The peptides from each sample were subsequently analysed using the TIMSTOF mass spectrometer (Bruker) in combination with the Evosep One system liquid chromatography (Denmark) operating in the DIA mode. The mass spectrometer recorded ion mobility MS spectra covering a mass range from m/z 100 to 1700. For accurate measurements, we carefully selected up to four windows, each corresponding to a 100 ms TIMS scan, based on the m/z-ion mobility plane. During PASEF MS/MS scanning, the collision energy was gradually increased from 20 eV at 1 /K0=0.85 Vs cm^−2^ to 59 eV at 1 /K0=1.30 Vs cm^−2^, following a linear ramp. The DIA data were also analysed using the Spectronaut 14.4.200727.47784 software by searching the previously constructed spectral library. The main software parameters included dynamic iRT for retention time prediction, enabled correction for interference on the MS2 level and enabled cross-run normalization. The results were filtered based on a Q value cut-off of 0.01, equivalent to an FDR (False Discovery Rate) of less than 1%.

### Integrated multi-omics analysis

To assess the relationship between proteomics data and microbial abundance, R software package ‘vegan’ was used to calculate the Bray–Curtis distance matrix for ‘proteomic data’ and ‘flora abundance’, respectively. Compare_distance_matrices.py (v 1.8.0) in QIIME software was used to conduct Mantel test statistical tests, and the samples were replaced (999 times) to evaluate the statistical significance (i.e. *P*-value) of the similarity between proteomics data and microbiome composition data. We employed the Mothur (version 1.41) [20] for calculating the Spearman ranking correlation coefficient. The correlation coefficient matrix revealed the degree of association between the two variables (rho correlation coefficient ranges from −1 to 1). When −1＜rho＜0, the two variables exhibit a negative correlation; on the other hand, when 0＜rho＜1, a positive correlation is observed. A rho value of 0 corresponds to no correlation between the variables. R package corrplot (version 0.92) was used for generating heat map [21]. Spearman’s correlation coefficient between proteomic data and microbial abundance was calculated. An association network was constructed for the related information of |rho|>0.8 and *P* value<0.01 and imported into Cytoscape software for visualization.

## Results and discussion

### Bacterial enumeration

**Fig. 1.**
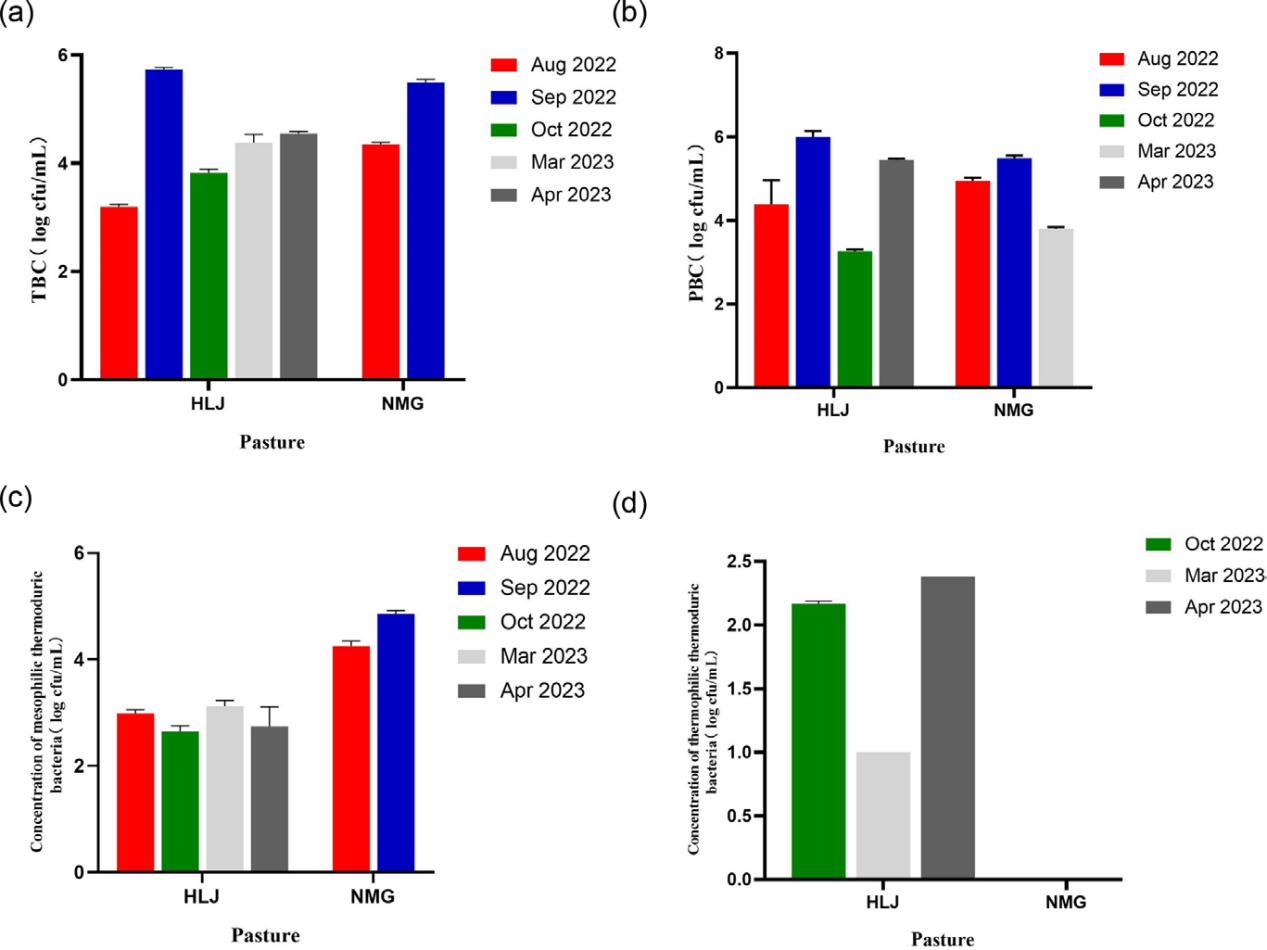
Bacterial counts of the different milk samples. (**a**) TBC, (**b**) PBC, (**c**) concentration of mesophilic thermoduric bacteria and (**d**) concentration of thermophilic thermoduric bacteria. The missing values represented no colonies were detected, and the error bars represented the error between three raw milk samples collected in the same group.

As shown in Fig. 1, the TBC varied between 3.15 and 5.76 log c.f.u. ml^−1^, with an average of 4.63 log c.f.u. ml^−1^ (4.34 log c.f.u. ml^−1^ for HLJ and 4.92 log c.f.u. ml^−1^ for NMG). The PBC ranged from 3.22 to 5.99 log c.f.u. ml^−1^, with a mean value of 4.82 log c.f.u. ml^−1^ (4.77 log c.f.u. ml^−1^ for HLJ and 4.87 log c.f.u. ml^−1^ for NMG). It should be noted that these findings align with the study conducted by Yang *et al.* [22]. The concentration of mesophilic thermoduric bacteria ranged from 2.38 to 4.9 log c.f.u. ml^−1^ with a mean value of 3.68 log c.f.u. ml^−1^ (2.87 log c.f.u. ml^−1^ for HLJ and 4.49 log c.f.u. ml^−1^ for NMG) and the concentration of thermophilic thermoduric bacteria ranged from 1 to 2.38 log c.f.u. ml^−1^ with a mean value of 1.81 log c.f.u. ml^−1^ for HLJ. Overall, TBC and PBC were higher in September 2022 compared to other months, and the concentration of thermophilic thermoduric bacteria was significantly higher in NMG than in HLJ. The HLJ group exhibited distinct thermophilic thermoduric bacteria that were not found in NMG.

Psychrotrophic bacteria and thermoduric bacteria play a vital role in assessing the quality of raw milk and the final dairy products. According to a study conducted by Yuan *et al.* [23], it has been observed that the presence of psychrotrophic and thermoduric bacteria in dairy products can lead to degradation in quality and shelf-life. These bacteria have the ability to produce enzymes, specifically proteolytic and lipolytic enzymes, which are capable of withstanding high temperatures during heat treatments. It is crucial to consider various factors such as the sanitation conditions of the milking environment, the overall health of the animals, the packaging methods and the handling practices to prevent the contamination of dairy products with these bacteria [24]. Furthermore, previous investigations have proven that the seasonal variations [25] and the geographical positioning [3] exert noteworthy influences on the community organization of psychrotrophic and thermoduric bacteria present in raw milk. Investigating the presence of psychrotrophic and thermoduric bacteria in raw milk is of great significance as it helps in understanding the sources of contamination and enables effective source control measures. According to Matta *et al.* [26], the threshold for milk spoilage to commence is ~10^6^ c.f.u. ml^−1^ in terms of PBC levels and the milk samples in September 2022 were close to the value (Fig. 1).

### Identification of psychrotrophic and thermoduric bacteria using culture-based method

The 16S rRNA PCR was conducted to determine the isolates at species or subspecies level [27]. Using the culture-based method, a total of 356 colonies were obtained from eight raw milk samples. These colonies consisted of 212 psychrotrophic bacteria and 144 thermoduric bacteria, displaying variations in their morphologies. To identify these isolates, sequencing of the 16S rRNA gene was performed. The psychrotrophic bacteria isolates exhibited a wide range of diversity, belonging to 26 different genera and 50 species. Similarly, the mesophilic thermoduric bacteria isolates were classified into 20 genera and 32 species. In contrast, the thermophilic thermoduric bacteria isolates were limited to only two genera and two species (Table 2). This extensive characterization of the bacterial communities in our samples emphasizes their varied composition and abundance of psychrotrophic and thermoduric bacteria. The dominant genera of psychrotrophic bacteria were *Pseudomonas*, *Lactococcus*, *Acinetobacter* and *Enterobacter*. Within the *Pseudomonas* species, *P. fluorescens* is renowned for its significant contribution to milk and dairy product spoilage [28]. Notably, most of *Pseudomonas* species were from samples of the dairy farm at NMG. *Lactococcus* and *Acinetobacter* were frequently isolated psychrotrophic genera, consistent with the findings from Bellassi *et al.*’s study [29]. The dominant genera of mesophilic thermoduric bacteria were *Enterococcus*, *Enterobacter*, *Lactococcus* and *Bacillus.* This result aligns with a previous study [3]. *Bacillus* has been widely reported as thermoduric bacteria, which can survive in high-temperature processing in the form of spores and can also produce hydrolase, resulting in spoilage of milk [30]. The thermophilic thermoduric bacteria have only been isolated from the dairy farm at HLJ. *Deinococcus geothermalis* and *Staphylococcus pasteuri* as thermophilic thermoduric bacteria have been isolated for the first time in this investigation. *D. geothermalis* was initially isolated from hot spring and runoff [31] and its thermoduric proteins, such as amylosucrase and novel thermostable single-stranded DNA-binding protein, have been effectively expressed for diverse applications in molecule biology [32]. *Staphylococcus* species are prominent pathogens within the mammary glands, frequently resulting in subclinical mastitis and sporadic clinical mastitis or enduring infection in dairy cows during lactation [33], which may come from cow’s teat, skin and unclean milking facilities and environment [34]. Our result suggests that *Enterococcus faecalis*, *Enterobacter cloacae*, *Enterobacter kobei*, *Enterobacter asburiae*, *Moraxella osloensis*, *Lactococcus lactis*, *Lactococcus raffinolactis* and *Leuconostoc pseudomesenteroides,* exhibiting psychrotrophic and thermoduric traits, should attract more attention.

**Table 2.** Identification and distribution of bacteria isolated from raw milk samples collected from dairy farm A and dairy farm B

Genus	Species	Total	No. of isolates		Genus	Species	Total	No. of isolates	
			HLJ	NMG				HLJ	NMG
Psychrotrophic bacteria					*Streptococcus*	*parauberis*	1	1	
*Chryseobacterium*	*bovis*	2	2		*Psychrobacter*	*pulmonis*	2	2	
	*manosquense*	2	2		*Pedobacter*	*gandavensis*	2		2
*Acinetobacter*	*guillouiae*	8	1	7	*Janthinobacterium*	*lividum*	2		2
	*parvus*	1		1		*agaricidamnosum*	1		1
	*albensis*	4		4	*Sphingobacterium*	*faecium*	1		1
	*harbinensis*	1		1		*anhuiense*	2		2
*Moraxella*	*osloensis*	3	3		Mesophilic thermoduric bacteria				
*Stenotrophomonas*	*maltophilia*	1		1	*Kocuria*	*varians*	10	2	8
*Klebsiella*	*oxytoca*	3	2	1		*salsicia*	5	5	
	*pneumoniae*	5	5		*Enterococcus*	*faecalis*	11	8	3
	*michiganensis*	1	1			*faecium*	1	1	
*Pseudomonas*	*azotoformans*	1		1	*Enterobacter*	*cloacae*	8	7	1
	*gessardii*	4		4		*kobei*	1	1	
	*fluorescens*	7		7		*asburiae*	1	1	
	*brenneri*	3		3	*Streptococcus*	*macedonicus*	1	1	
	*kairouanensis*	1	1			*thermophilus*	4	4	
	*jessenii*	1		1	*Microbacterium*	*lacticum*	8	3	5
	*libanensis*	1		1	*Moraxella*	*osloensis*	8	5	3
	*cedrina*	2		2	*Agrobacterium*	*tumefaciens*	1		1
	*veronii*	1		1	*Acinetobacter*	*nosocomialis*	1	1	
	*helvolus*	1		1	*Salmonella*	*enterica*	2		2
*Pseudoclavibacter*	*terrae*	1	1		*Brachybacterium*	*nesterenkovii*	2		2
*Enterococcus*	*faecalis*	11	10	1	*Proteus*	*mirabilis*	6		6
*Enterobacter*	*kobei*	9	9		*Gordonia*	*paraffinivorans*	1		1
	*asburiae*	1		1	*Sphingobacterium*	*mizutaii*	1		1
	*huaxiensis*	4	4		*Chryseobacterium*	arthrosphaerae	3		3
	*cloacae*	14	9	5		*carnipullorum*	1		1
	*sichuanensis*	2	2		*Staphylococcus*	*saprophyticus*	1		1
*Microbacterium*	*maritypicum*	1		1		*capitis*	1		1
*Epilithonimonas*	*bovis*	5	4	1	*Lactococcus*	*lactis*	32	32	
	*vandammei*	1		1		*raffinolactis*	10		10
*Lactococcus*	*raffinolactis*	36	35	1		*cremoris*	1	1	
	*lactis*	6	1	5	*Lactobacillus*	*paracasei*	2	2	
*Leuconostoc*	*pseudomesenteroides*	4	4		*Leuconostoc*	*lactis*	1	1	
	*mesenteroides*	7	7			*pseudomesenteroides*	1	1	
*Pantoea*	*agglomerans*	1	1		*Bacillus*	*amyloliquefaciens*	10	10	
*Macrococcus*	*caseolyticus*	1		1		*cereus*	4	4	
*Comamonas*	*piscis*	3	3			*tropicus*	1	1	
*Kluyvera*	*intermedia*	28	28		*Rothia*	*kristinae*	2	2	
*Lelliottia*	*amnigena*	6	6		Thermophilic thermoduric bacteria				
*Carnobacterium*	*maltaromaticum*	4	2	2	*Deinococcus*	*geothermalis*	1	1	
*Corynebacterium*	*callunae*	2		2	*Staphylococcus*	*pasteuri*	1	1	
*Comamonas*	*piscis*	1		1					

### Composition of psychrotrophic and thermoduric bacterial microbiota

The results of sequencing depth and quality are summarized in Additional File S1. A total of 420 million reads and 63.48 billion bases were generated from the samples, yielding an average of 70.07 million reads and 10.58 billion bases per sample. The base identification accuracy exceeded 97%, reaching over 99%. The CleanData set contained an average of 491143 sequences. Furthermore, the assembly and splicing processes resulted in an average of 2627 scaffold sequences, with an N50 value of 708. These results indicated a successful splicing effect and suggested that the obtained metagenomic sequences are relatively complete.

**Fig. 2.**
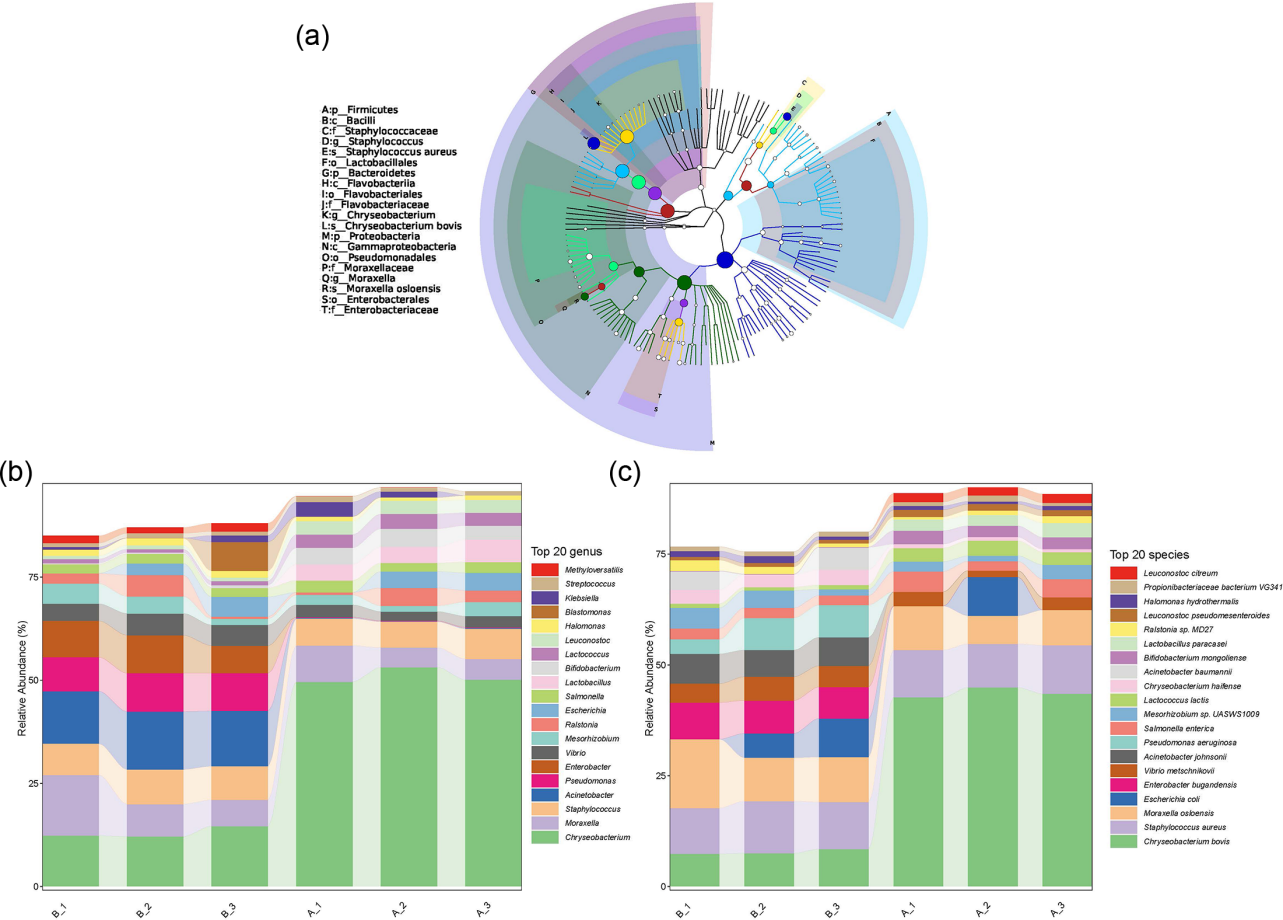
Composition of psychrotrophic and thermoduric bacterial microbiota. (**a**) Classification tree of raw milk samples based on GraPhlAn. The species and phylum are ordered from the inner circle to the outer circle, and the size of the node represents the average relative abundance of all taxa. (**b**) The genus level and species level include the top 20 taxa.

Based on the results of microbial enumeration, it was evident that the contamination in September 2022 was more severe compared to other months (Fig. 1). Consequently, the metagenomic investigation concentrated on the two groups of samples obtained from HLJ and NMG, designated as A and B, specifically from September 2022. Fig. S1 (available in the online version of this article) depicts the rarefaction curves, indicating that the sequencing depth adequately anticipated the diversity found in the samples. The classification class tree of micro-organisms in raw milk generated by GraPhlAn is shown in Fig. 2a. Proteobacteria was the main group of psychrotrophic bacteria in raw milk, followed by Firmicutes and Bacteroidetes. The bacterial genus included *Chryseobacterium*, *Staphylococcus* and *Moraxella* with *Staphylococcus aureus*, *Chryseobacterium bovis* and *M. osloensis *main species. The dominant species in each sample were found at each classification level, as shown in Fig. 2b. The abundance of the bacteria *C. bovis* varied between the two dairy farms, whereas the abundance of *M. osloensis* and *S. aureus* was similar. *M. osloensis* was identified during the screening of psychrotrophic and thermoduric bacteria, underscoring its environmental adaptability. *L. lactis* was detected as both psychrotrophic and mesophilic thermoduric bacteria. Other psychrotrophic bacteria identified, included *Acinetobacter johnsonii* and *L. pseudomesenteroides*.

### Variance of bacterial diversity between locations

**Fig. 3.**
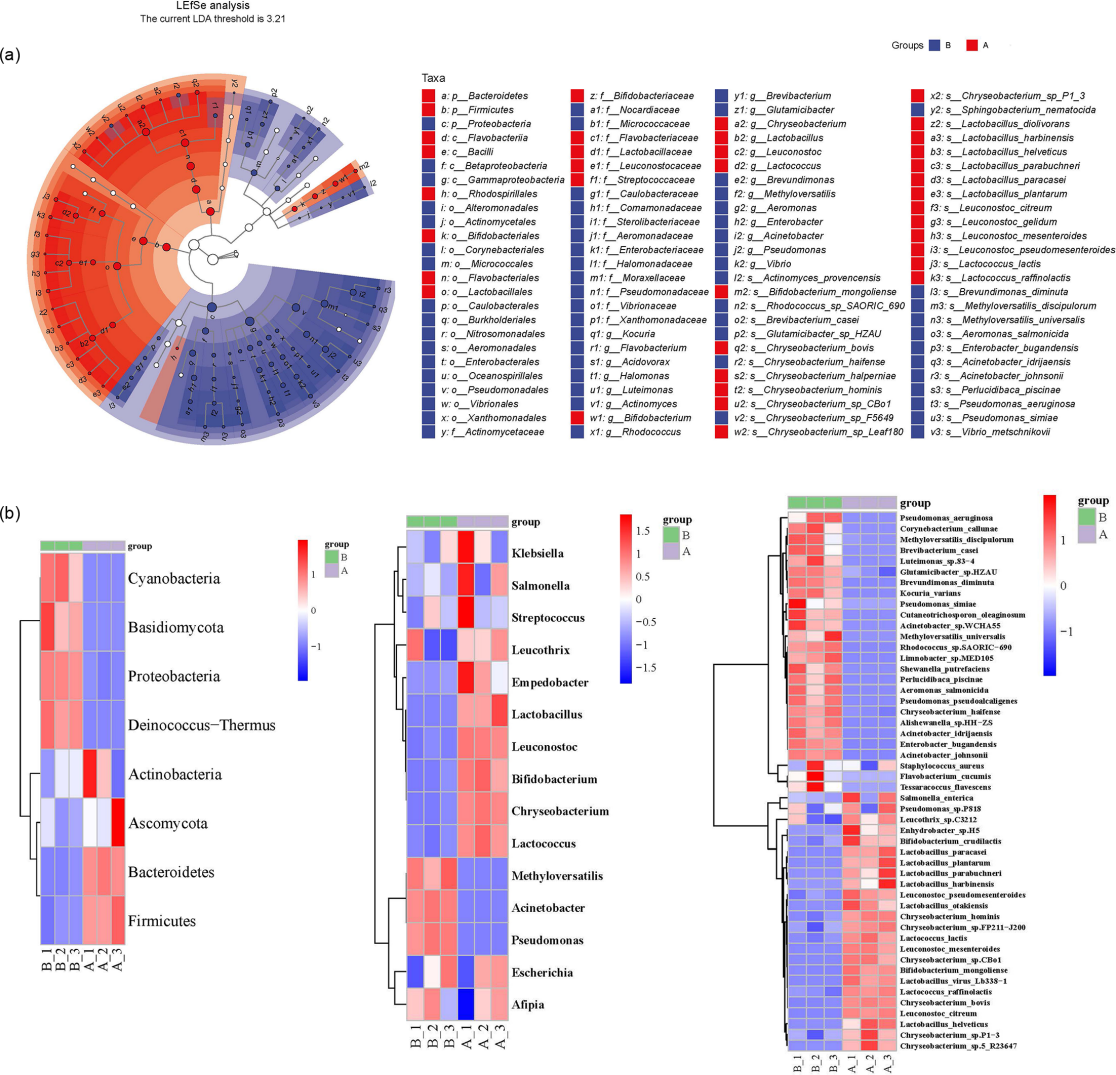
Bacterial diversity differs across various locations. (**a**) The bacterial community structure in raw milk samples was analysed using LEfSe. Significant differences in taxa are indicated by blue or red nodes. Taxa with insignificant differences are represented by hollow nodes. The size of each node corresponds to the relative abundance of the taxa. (**b**) A clustering heat map was generated to compare families, genus and species of raw milk samples collected from dairy farm A and dairy farm B.

Through LEfSe analysis, 103 taxa were observed, exhibiting significant distinctions between groups A and B (Fig. 3a). Specifically, 37 taxa showed a substantial prevalence within group A, while 66 taxa displayed a considerable prevalence within group B. The top 50 taxa with significant differences in relative abundance between the samples were presented by cluster analysis and heat map (Fig. 3b). The analysis revealed that the sample replicates were clustered together, indicating good research consistency. The community profiles were significantly distinct between two dairy farms. *Methyloversatilis*, *Acinetobacter* and *Pseudomonas* were the dominant genus in group B. *Leucothrix*, *Empedobacter*, *Lactobacillus*, *Leuconostoc*, *Bifidobacterium*, *Chryseobacterium* and *Lactococcus* were the dominant genus in group A. The PCoA plot (Fig. S2) supported the observed differences.

The study conducted by Du *et al.* [35] aimed to explore the species diversity of psychrotrophic bacteria found in raw milk collected from various provinces in China, including Heilongjiang, Anhui, Jiangsu, Inner Mongolia, Gansu, Chongqing, Henan and Hunan. They found that the microbial compositions of Heilongjiang and Inner Mongolia were similar to other cities, with *Pseudomonas*, *Stenotrophomonas* and *Enterococcus* being highly isolated, which aligns with our study findings. Liang *et al.* [36] found that *Pseudomonas* was the most abundant bacteria genus, followed by *Lactococcus* and *Acinetobacter* by using single-molecule real-time sequencing technology for raw milk from five provinces in China. Another research on mesophilic bacterial diversity in raw milk from southern Germany showed that *Staphylococcus*, *Streptococcus* or *Janibacter*, *Chryseobacterium* and *Acinetobacter* were the main bacterial genera by pure culture and high-throughput sequencing.

### Metaproteomic investigation of the microbial communities

**Fig. 4.**
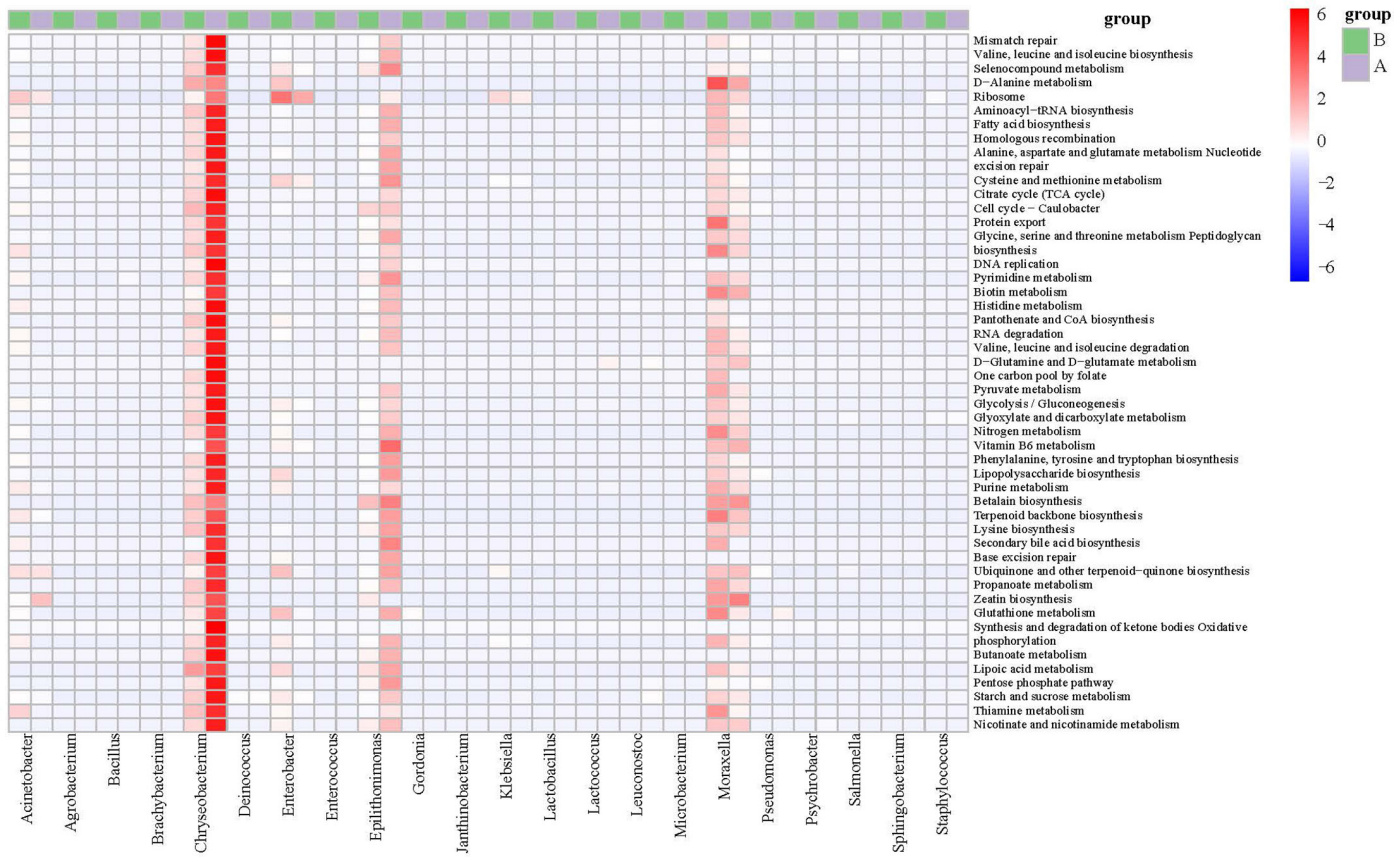
Functional characterization of the microbiota of raw milk samples collected from dairy farm A and dairy farm B. Heatmap illustrates biological processes enriched in the microbiota of the two groups of milk samples. The colour scale represents relative protein abundance.

To assess the potential impacts of psychrotrophic and thermophilic bacteria on raw milk, we classified proteins using the KO database and completed functional characterization of the milk microbiota (Fig. 4). Overall, *Chryseobacterium*, *Moraxella* and *Epilithonimonas* could be key bacteria in raw milk. These bacteria were characterized as psychrotrophic bacteria, with *Chryseobacterium* and *Moraxella* also being classified as thermoduric bacteria. Various pathways, including glycolysis/gluconeogenesis, citrate cycle (TCA cycle), ascorbate and aldarate metabolism, ribosome, RNA transport, RNA degradation, RNA polymerase, pentose phosphate pathway, DNA replication and mineral absorption, were identified facilitating microbial growth and metabolism in raw milk. The activation of pathways, such as two-component system, quorum sensing, lipopolysaccharide biosynthesis and peptidoglycan biosynthesis pathways, was observed, indicating their role in supporting the stress resistance of microbial community and enabling them to adapt to the changing environment [37]. The protein analysis detected various proteins associated with the bacterial secretion system, proteasome, protein export, biosynthesis of unsaturated fatty acids and protein digestion and absorption, suggesting the presence of potential proteases and lipases in the microbial community.

The analysis of the microbiota revealed the presence of 36 proteins and 58 peptides in both sample groups through metaproteomics. A notable dissimilarity was observed in the bacterial protein profiles between the two groups of samples, as indicated by the principal component analysis (PCA) (Fig. S3a). There were three unique proteins distinguished from the two groups (Fig. S3b). The functions of 36 proteins were determined, as shown in Additional File S1. The functional characterization of the milk microbiota was classified by utilizing diverse protein ontology databases, which encompassedKO, eggnoggNOG, Swissprot, Cazyme and MEROPS. The functional classification overview is available in Additional File S1. A large number of peptidases were annotated, such as PepA aminopeptidase, aminopeptidase P3, HtpX peptidase and AlgW peptidase, which mainly came from *Chryseobacterium*, *Epilithonimonas*, *Pseudomonas*, *Psychrobacter*, *Acinetobacter, Lactococcus, Escherichia* and *Bacillus*. These results have been approved by many studies. PepA aminopeptidase has been reported in *Escherichia coli* [38], *L. lactis* [39] and *Tetragenococcus halophilus* [40]. Aminopeptidase P was identified from *L. lactis* previously [41]. *E. coli*’s HtpX peptidase is recognized as a cytoplasmic membrane zinc metalloproteinase belonging to the M48 family, whose function revolves around maintaining the quality control of membrane proteins [42]. Although none of the above peptidase was detected by metaproteomics, the potential of specific bacteria in raw milk to produce hydrolytic peptidase has been confirmed. We speculated that the phenomenon was attributed to non-expression or little expression of the relevant proteolytic and lipolytic genes in fresh raw milk.

### Multi-omics analysis of microbial communities and identified proteins

**Fig. 5.**
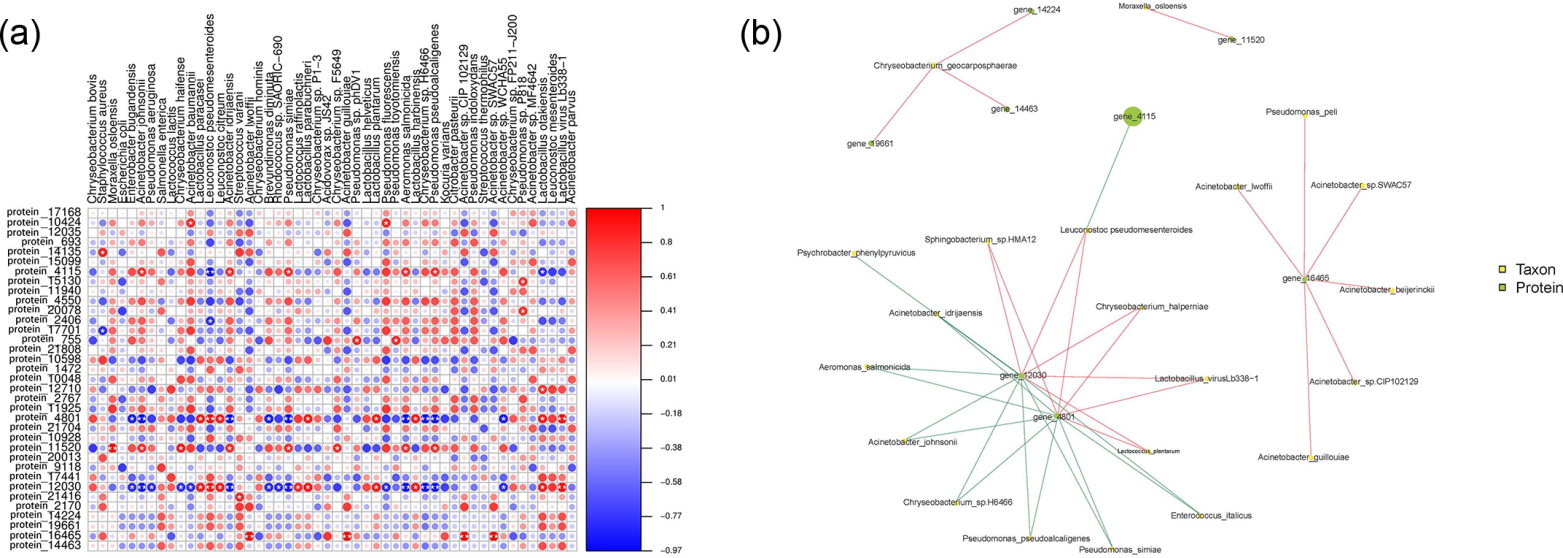
Correlogram analysis of identified proteins and bacteria. (**a**) Association of heat-map analysis. Mantel test was conducted on a sample group level (**a and b**) to emphasize the factors contributing to the statistically significant differences. Statistical significance is denoted by single and double asterisks representing *P*<0.05 and *P*<0.01, respectively. (**b**) Network diagram of correlation. The links connecting nodes illustrate correlation, wherein the red line denotes a positive correlation and the blue line denotes a negative correlation. The greater the number of connections lining up with a node, the more microflora information it is associated with. The size of the node area corresponds to the higher value of the species abundance/detection index represented by that node.

In order to work out the expression and function of psychrotrophic and thermoduric bacteria protein in fresh raw milk, the data from metagenomics and metaproteome were further integrated. The identified proteins were discovered to be linked with various psychrotrophic bacteria (including *C. bovis*, *Acinetobacter guillouiae*, *Pseudomonas* spp. and *Leuconostoc mesenteroides*), thermoduric bacteria (including *Lactobacillus paracasei*, *Kocuria varians* and *Streptococcus macedonicus*), and psychrotrophic/thermoduric bacteria (such as *M. osloensis*, *L. lactis*, *L. pseudomesenteroides*, *L. raffinolactis*, *Chryseobacterium* spp. and *Acinetobacter* spp.). Fig. 5 shows the association between differentially abundant bacteria and expressed protein. The relative expression abundances of protein 10424, 15130, 20078, 755, 11520 and 11465 were high, whereas proteins 4115 and 2406 were low (Fig. 5a). According to Fig. 5b, the majority of microflora was correlated with small ribosomal subunit protein uS15 (protein 12030), malate dehydrogenase (protein 4801) and nucleoside-diphosphate kinase (NDPKs; protein 16465). Protein 4115 showed great species abundance, indicating a high level of protein expression. The CAZyme classified it as Glycoside Hydrolase Family 36 (GH36), which serves as U Intracellular trafficking, secretion and vesicular transport. GH36 exhibits *α*-galactosidase and *α*-*N*-acetylgalactosaminidase activity, firstly reported in an NMR study on the *α*-galactosidase GalA from *Thermotoga maritima* [43]. It involves the formation of a covalent glycosyl-enzyme intermediate [44]. We speculated that *L. pseudomesenteroides* may produce extracellular enzymes and result in lactose hydrolysis [45]. Protein 16465 was identified as NDPKs derived from *Acinetobacter* sp. (Fig. 5b) and Additional File S1. Additionally, Protein 16465 was associated tightly with *Pseudomonas peli*, indicating *Pseudomonas* spp. may play a role in promoting the protein expression of *Acinetobacter* spp. NDPKs found in the study are evolutionarily conserved enzymes that catalyse phosphoryl transformation between nucleosides [46]. In bacteria, NDPKs appear as dimers or tetramers [47], showing important status in DNA and RNA biosynthesis. In addition, it actively participates in the synthesis of polysaccharides and proteins, while also playing a crucial role in energy metabolism. Protein 12030 was identified as AA4 by CAZyme annotation. The VAO (Vanillyl alcohol oxidase) enzymes, found in the AA4 family, perform the important role of facilitating the transformation of phenolic compounds that possess side chains located at the para-position of the aromatic ring. The EggNOG and Nr databases have assigned J as the annotation for Protein 12030, which is associated with translation, ribosomal structure and biogenesis. Similarly, CAZyme has annotated Protein 4801 as GH3. The Glycoside Hydrolase Family 3 category includes a variety of enzymes, such as exo-acting *β*-d-glucosidases, *α*-l-arabinofuranosidases, *β*-d-xylopyranosidases, *N*-acetyl-*β*-d-glucosaminidases (glycoside hydrolases) and *N*-acetyl-*β*-d-glucosaminide phosphorylases. One specific *N*-acetyl-*β*-d-glucosaminidase belonging to the GH3 family, known as NagZ, has been reported to play a role in bacterial cell wall recycling [48]. In Gram-negative bacteria, NagZ removes GlcNAc from 1,6-anhydroMurNAc-peptides [49], whereas in Gram-positive bacteria, it removes GlcNAc from GlcNAc-MurNAc-peptides [50]. According to Mark *et al.* [51], the NagZ product, which is known as 1,6-anhydroMurNAc-peptide, plays a crucial role in promoting the overproduction of AmpC β-lactamase in various Gram-negative micro-organisms. This particular compound has garnered attention as a potential target for therapeutic interventions [51]. Protein 4801, annotated as C Energy production and conversion by eggnog and as malate dehydrogenase by KO and Nr, participates in diverse bioprocesses. These include the citrate cycle, metabolism of cysteine and methionine, pyruvate metabolism, glyoxylate and dicarboxylate metabolism, methane metabolism in different environments and the carbon metabolism pathway. Based on the aforementioned protein functions, a significant portion of the identified proteins was closely associated with bacterial growth and adaptation to the environment.

## Conclusions

In this study, significant variations were observed in the microbial composition of raw milk samples across different sampling months and locations, indicating a notable diversity of psychrotrophic and thermoduric bacteria in the raw milk samples that were tested. The microbial compositions of psychotrophic and thermoduric bacteria were significantly distinct between the two dairy farms, specifically in the month of September 2022. The majority of proteins detected in the proteomic profiling were indicative of bacterial growth at an early stage, in strong association with bacterial growth, reproduction and adaptation to cold environments. However, the functional genes related to microbial hydrolases, proteases and lipases remained undetectable. A hypothesis proposes that the concentration of spoilage enzymes is anticipated to rise when the growth concentration surpasses a specific threshold. The comprehensive analysis through metagenomic and proteomics in our study offers valuable insights to better understand the potential impact of the microbial content influencing milk quality. Further investigation will be conducted to explore the potential impact of heat-resistant enzymes generated by psychrotrophic and thermoduric bacteria on the quality of milk.

## supplementary material

10.1099/mgen.0.001311 Uncited Supplementary Material 1.

10.1099/mgen.0.001311 Uncited Supplementary Material 2.

## Abbreviations

AGE: agarose gel electrophoresis
CAZy: carbohydrate-active enzymes
DDA: data-dependent acquisition
DIA: data-independent acquisition
DIA: data-independent acquisition
EggNOG: Evolutionary Genealogy of Genes: Non-supervised Orthologous Group
FDR: false discovery rate
HLJ: Heilongjiang
IAA: iodoacetic acid
iRT: indexed retention time
KEGG: kyoto encyclopedia of genes and genomes
KO: KEGG orthology
LB: luria bertani
LEfSe: linear discriminant analysis effect size
MPC: milk powder count agar
NDPKs: nucleoside-diphosphate kinase
NMG: Inner Mongolia Province
ORFs: open reading frames
PASEF: parallel accumulation–serial fragmentation
PBC: psychrotrophic bacterial count
PCA: plate counting agar
PcoA: principal coordinate analysis
PCR: polymerase chain reaction
SDT: SDS and DTT
SMRT: single molecule real-time
SRA: sequence read archive
SSB: single-stranded DNA-binding
TBC: total bacterial count
TCA: citrate cycle
TIMSTOF: trapped ion mobility spectrometry time of flight
VAO: vanillyl alcohol oxidase
