# Supplementary material for: Characterization of psychrotrophic and thermoduric bacteria in raw milk using a multi-omics approach
Source: Microb Genom. 2024 Nov 6;10(11):001311. doi: 10.1099/mgen.0.001311 (PMC11540130; doi:10.1099/mgen.0.001311)
Supplement: Uncited Supplementary Material 2. [file mgen-10-01311-s002.pdf]

SUPPLEMENTARY MATERIALS

File S1 Function of identified proteins and MEROPS annotation with taxonomy based on metagenome (uploaded as excel)

Fig.S1 Rarefaction curves of Shannon's diversity index for raw milk samples collected from dairy farm A and dairy farm B.

Fig.S2 Principal coordinates analysis (PCoA) of the bacterial community structure from raw milk samples collected from dairy farm A and dairy farm B

Fig.S3 Metaproteomics data. a) Analysis of the bacterial proteins in raw milk samples obtained from dairy farm A and dairy farm B using PCA, showcasing the distinct division between the two groups based on the identified proteins. b) The number of proteins identified in raw milk samples from dairy farm A and dairy farm B through a Venn diagram.

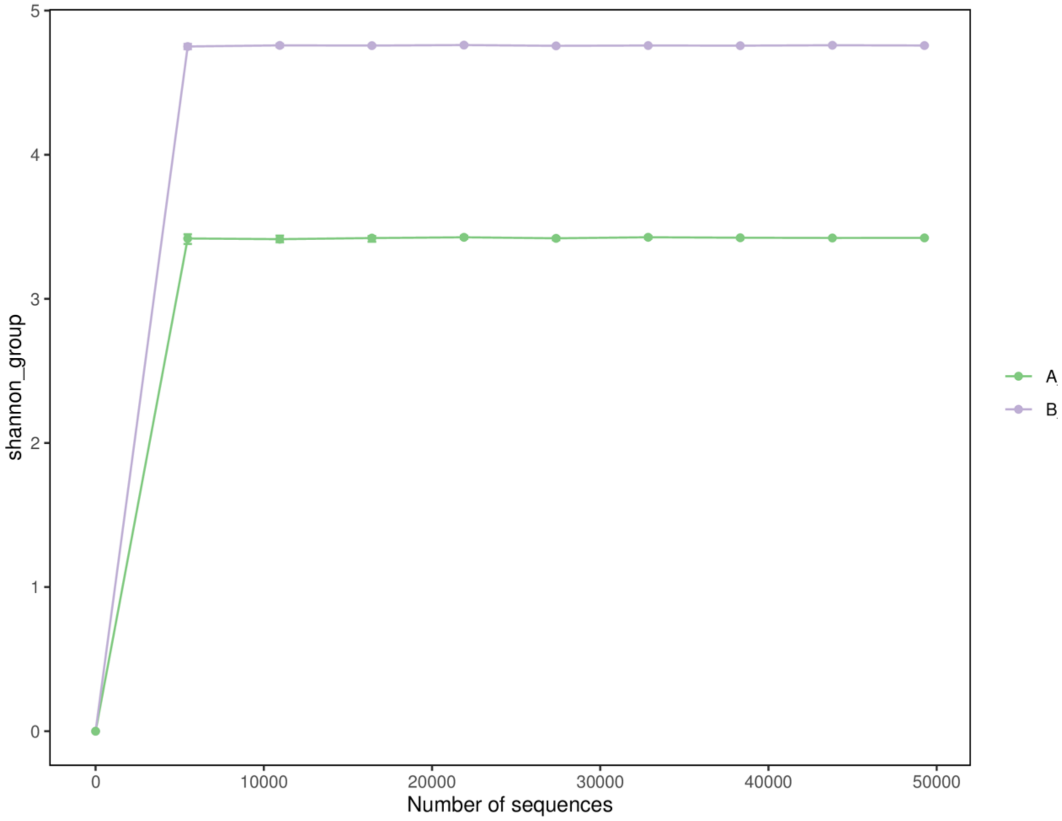

shannon_group
Number of sequences
0
1
2
3
4
5
0
10000
20000
30000
40000
50000
A
B

Fig.S1

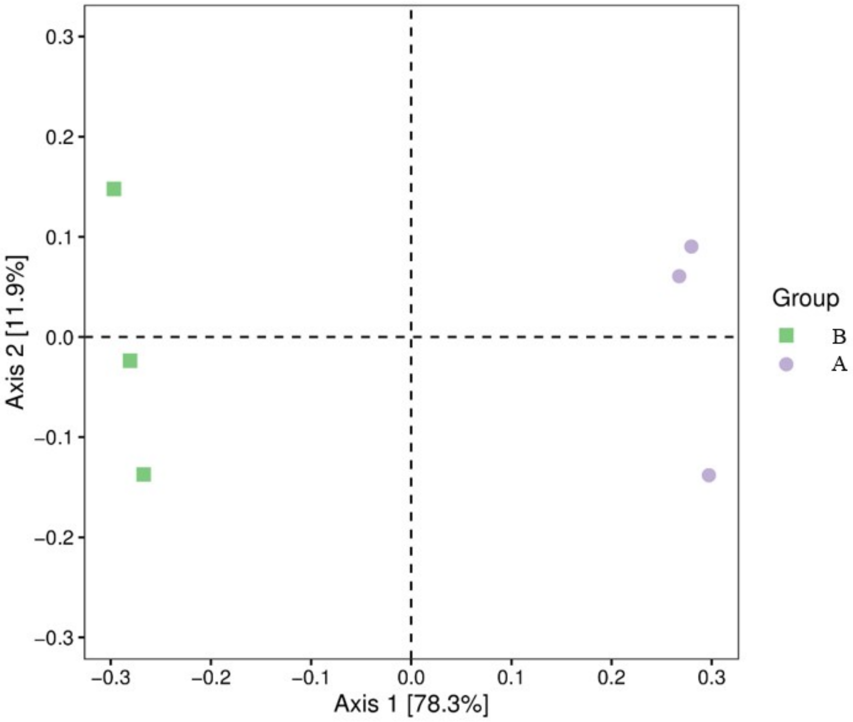

Axis 2 [11.9%]
Axis 1 [78.3%]
0.3
0.2
0.1
0.0
−0.1
−0.2
−0.3
−0.3
−0.2
−0.1
0.0
0.1
0.2
0.3
Group
B
A

Fig. S2

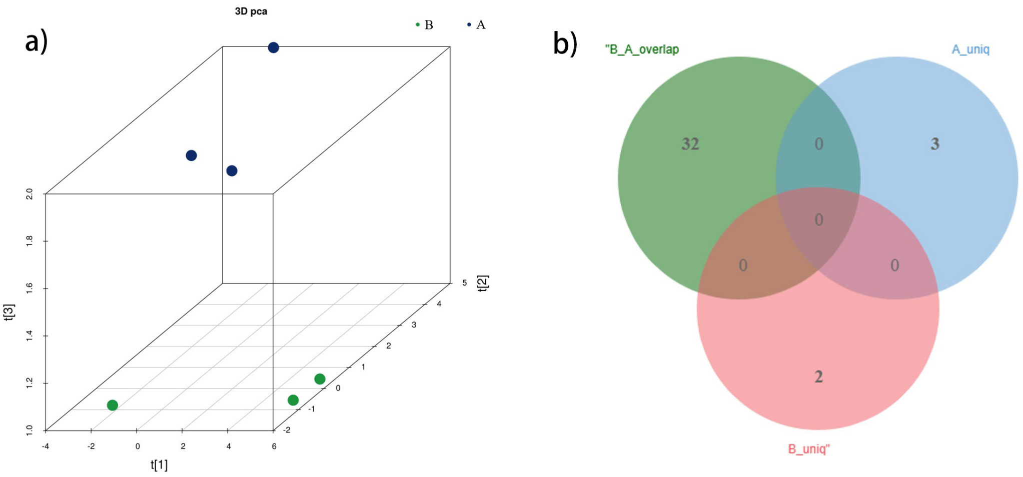

Fig.S3
